# The Law of Minimum Vertical Dimension: Evidence for Improvement of Dental Occlusion

**DOI:** 10.1055/s-0041-1732950

**Published:** 2021-10-21

**Authors:** Silvana Silveira, Patricia Valerio, Almiro J. Machado Júnior

**Affiliations:** 1 Functional Jaw Orthopedics, Cascais, Portugal; 2 Patricia Valério Institute, Belo Horizonte, Brazil; 3 Department of Otorhinolaryngology, Head and Neck Surgery, University of Campinas, Campinas, São Paulo, Brazil

**Keywords:** centric relation, maximum intercuspation, occlusal plane, malocclusion, neuro-occlusal rehabilitation, vertical dimension, unilateral chewing

## Abstract

The law of minimum vertical dimension (MVD) states that “when the mandible moves to reach the maximum intercuspal position, this always involves bringing the mandible and maxilla as close together as possible.” Therefore, after the first occlusal contact is made, the MIP will be reached through reduction of the vertical dimension. Our objective of this study, through an integrative review of the literature review, was to determine whether ignoring this law is a factor that contributes to malocclusion, temporomandibular joint dysfunction, and recurrences of functional orthodontic and orthopedic treatments.We conducted a search of the literature in five of the main electronic scientific databases. The following medical subject heading terms were used in our search: centric relation, dental occlusion, malocclusion, vertical dimension, and mastication. We cross-referenced the descriptors in the following four groups: centric relation and maximum intercuspation; occlusal plane and malocclusion; neuro-occlusal rehabilitation; and vertical dimension and unilateral chewing. From this, we selected 277 potentially eligible articles. Out of these, 209 were excluded in accordance with the exclusion criteria already described. Thus, 65 studies were included in the qualitative synthesis.The articles were also classified according to their impact factor and degree of recommendation, in conformity with the table of the Oxford Centre for Evidence-Based Medicine. The scientific interest in the scope of the articles was also assessed by using three charts developed according to year and country of publication and the percentage of publication. Unilateral chewing creates a vicious cycle of damage that leads to an ever-increasing masticatory deficiency. Most of the articles chosen for this review confirmed that noncompliance with law of MVD was a predisposing factor in cases of relapse, in functional orthodontic and orthopedic treatments, as well as a causal factor in malocclusion and in functional and morphological TMJ dysfunctions.

## Introduction

One of the fundaments of functional jaw orthopedics, from a neuro-occlusal rehabilitation (NOR) perspective, is knowledge of existing laws and theories regarding craniofacial growth and development. 1 2 The NOR philosophy, as conceived by Planas, 3 can be defined as the part of dentistry in which the causes and the beginning of functional and morphological dysfunctions of the stomatognathic system are studied. Its goal is to investigate and eliminate their underlying causes and, whenever possible, to rehabilitate the patient or reverse the damage. 3 4 5 Planas 3 observed that bilateral mastication taking place on alternating sides and free from occlusal interference, with as many points of contact as possible during the masticatory cycles, conditions correct development of both the mandible and the maxilla. This confirms Claude Bernard's principle that “the function creates the organ and the organ adapts to the function.” 6 7 Therefore, since mastication is one of the craniofacial development factors, it must be done on hard dry foods, that is, those that give rise to intensive work, with broad lateralized movement and as many physiological dental contact events as possible, thus obtaining greater efficiency. 8 9 The complexity of masticatory movements and their control and adaptability show the extent to which their variation can influence not only dental-alveolar growth, but also maxillary-mandibular growth, with adaptation of structural morphology to working conditions. 8

Furthermore, Planas 3 studied and described the physiology of masticatory function. He drafted a set of laws, better known as Planas' Development Laws, as follows: anteroposterior and transverse development law, vertical premolar and molar development law, vertical incisor development law, occlusal plane development law, and minimum vertical dimension (MVD) law. 3 10 The law of MVD forms the scope of the present study and states that “when the mandible moves to reach the maximum intercuspal position (MIP), this always involves bringing the mandible and maxilla as close together as possible.” This means that after the first occlusal contact is made, the MIP will be reached through reduction of the vertical dimension (VD). 3 4 7 11 12 13

In the resting position (RP), that is, the position without physical tooth contact, there is free interocclusal space, and the condyles sit at their uppermost and frontmost position in the joint cavities. 13 From this RP, with mouth closure as far as the first occlusal contact, there is a reduction of the VD for the lower third of the face. This position is the centric occlusion (CO) and it may coincide with the MIP. In this case, the CO will be the functional occlusion (FO). This would constitute a case of normal physiological occlusion. 1 3 4 9 14 15

The FO establishes the maximum intercuspal contact between the upper and lower dental arches. Any lateral or protrusive excursion of the jaw starting at that point will cause an increase in the VD, even if infinitesimally small. If the CO is different from the MIP, however the jaw could shift toward the MIP, and that side with the smallest VD will have the FO. 16 17 18 19 20 21 An equal VD in the transverse and/or sagittal directions means that the patient possesses the mechanical conditions to perform free, ample, and bilaterally alternating mastication. On the other hand, if there are different increases in VD on each side during the functional excursive motion of the jaw, it can be suggested that the person will chew on the side where the increase is smaller, that is, the MVD side. In this case, one of the condyles will always be located outside the bottom of the joint cavity. 22 23 24 25 26 27 28 29 30 31

Moreover, if there is a difference in disocclusion angles (PMFA), the individual will remain with or will develop unilateral chewing, thus compromising the stability of the malocclusion correction over the long term.

**Fig. 1:**
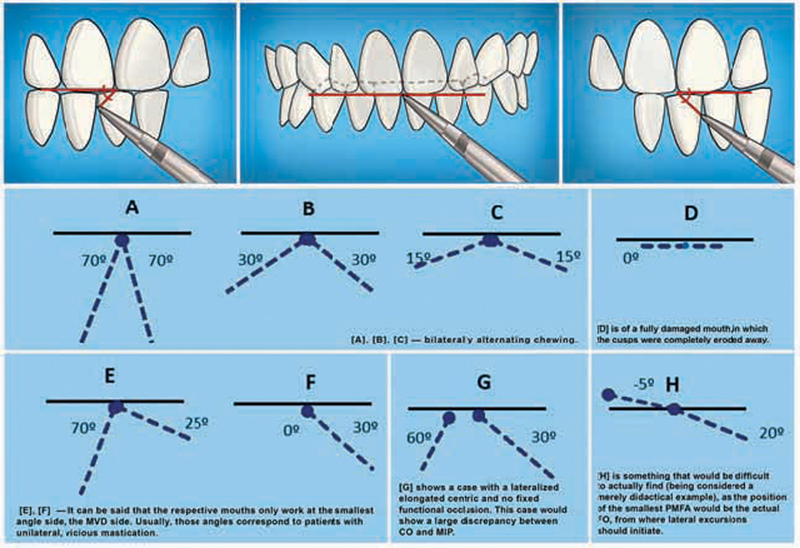
Planas' masticatory functional angle (image modified from reference 3 ).

To observe the patient's jaw motion, Planas 3 defined an angle, which is described by the end trajectory of the mastication cycles: the Planas masticatory functional angle (PMFA). This angle is defined by the increases in the left-side and right-side VDs and the horizontal plane during lateral motion. 7 8 12 15 During functional jaw motion, it is possible to check whether the jaw motion is bilaterally equal or not by placing a marker on the lower interincisor point, just underneath the upper incisor's incisal edge, and asking the patient to move the jaw without disconnecting the dental contacts on each side. 10 These angles are the visual representation of the lateral jaw motion ( Fig. 1 ). When mastication is physiological, that is, bilaterally alternating, the right and left PMFAs are equal, thus leading to an equivalent and symmetrical increase in lateral VD. 3 4 7 8

On the other hand, if mastication is pathological, these angles are unequal, the increase in the lateral VD is larger on one side than on the other, and chewing takes place on the side where the PMFA is lower. 12 It therefore seems pertinent to point out that to achieve balance in the stomatognathic system, subsequent to stabilization through orthodontic treatment, it is crucial to assess both static and dynamic occlusion. During this assessment, it needs to be verified that the de-occlusion patterns (laterality or protrusion) do not present any deleterious contacts or unequal de-occlusion angles (PMFA). It also needs to be verified that bilateral chewing exists, 29 since lack of lateral jaw motion leads to an “opening-and-closing” chewing function, or to unilateral function with a preferred side for mastication. With chewing patterns of this nature, there is insufficient stimulus for craniofacial growth, potentially leading to damaged skeletal, muscle, and tooth positioning and unbalancing of the stomatognathic system. However, in some cases, functional adaptation to the malocclusion may arise, thus enabling uninconvenienced chewing, through compensatory mechanisms. 3 10 11

Considering that protrusive lateral movements of the mandible and adequate occlusal impact are fundamental preconditions for continuous adaptation to functional demands, our objective with this study, through an integrative review of the literature, was to determine whether ignoring the law of MVD is a factor that contributes to malocclusion, temporomandibular joint dysfunction (TMD), and recurrences of functional orthodontic and orthopedic treatments.

## Methods

We conducted a search of the literature in five of the main electronic scientific databases in December 2020 and January 2021. These databases were PubMed Central (PMC), Google Scholar, Medline/PubMed, Scientific Electronic Library Online Brazil, and Virtual Health Library/Latin American and Caribbean Health Sciences Literature. They were chosen because they allow searches with established criteria. The inclusion criteria were the studies that could be case–control studies, reviews, case reports, or randomized studies in the following languages: Portuguese, English, French, and Spanish.

The following Medical Subject Heading terms were used in our search: centric relation, dental occlusion, malocclusion, vertical dimension, and mastication.

Full-text articles were retrieved through CAPES (the scientific article search portal of Brazil's Coordination Office for Improvement of Higher-Education Personnel), PMC database access, ResearchGate, and Google Scholar, where some of the articles were available. The bibliographic references of the articles selected were also assessed, and those considered pertinent were also included in this study.

Searches for articles according to author name, using the names of renowned authors in this field, and in the gray literature were also conducted, and these comprised 19% of the articles selected.

To request missing or additional data, or to clarify certain information, we contacted the corresponding authors of the respective articles through ResearchGate or via e-mail, applying a standardized e-mail template. Article selection took place after applying the following exclusion criteria: articles about syndromes (given that pathological conditions were not within the scope of this review); articles about diet/nutrition with no correlation to craniofacial development or occlusion; and animal studies (given that animals are considered to be “biological reagents” and that experimental results can be skewed by the environmental, genetic, and experimental circumstances of the species used). Foreign (non-Portuguese) language publications were translated by English language translators.

**Fig. 2:**
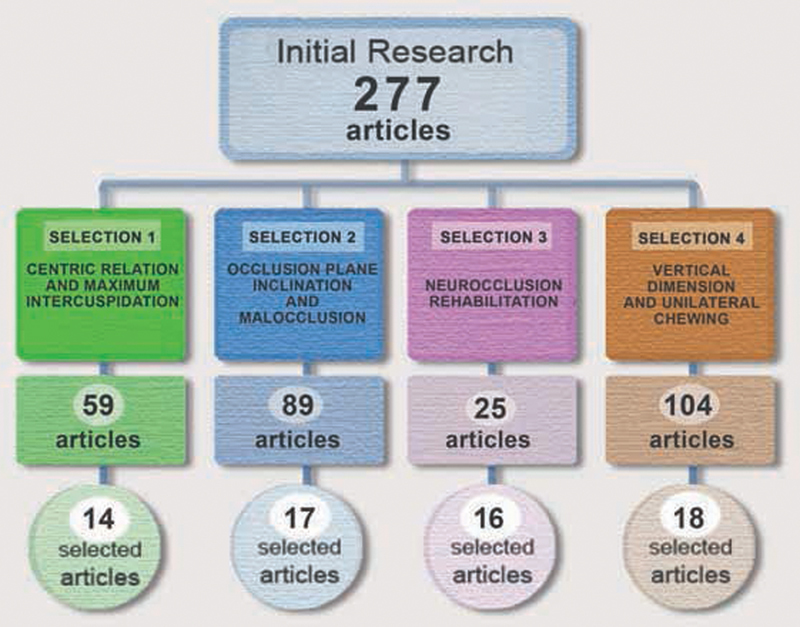
Flow chart.

We cross-referenced the descriptors in the following four groups: centric relation and maximum intercuspation; occlusal plane and malocclusion; NOR; and vertical dimension and unilateral chewing. From this, we selected 277 potentially eligible articles. Out of these, 209 were excluded in accordance with the exclusion criteria already described. Thus, 65 studies were included in the qualitative synthesis, as shown in the flowchart ( Fig. 2 ).

**Fig. 3:**
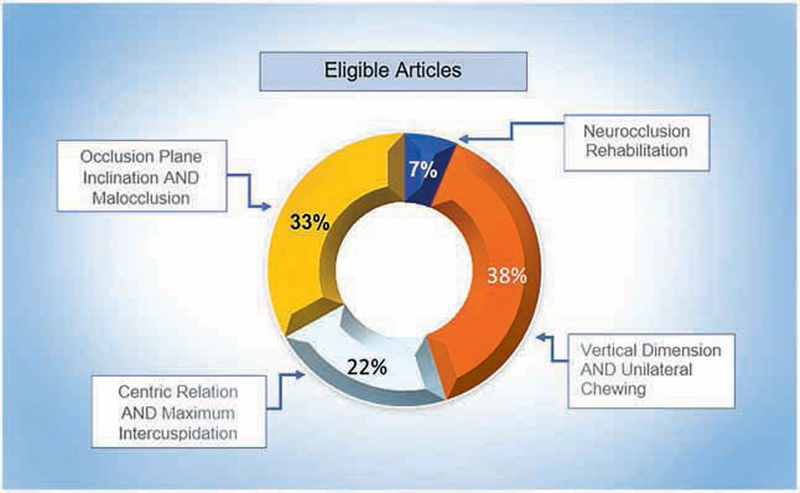
Eligible articles.

Even though one of the basic principles of evidence-based practice is that review of scientific publication should be contemporary, with approximately 10 years as a timeframe, we extended the search parameters to encompass the past 20 years from January 1, 2000 to December 31, 2020. We did this because although the literature that provides the scientific basis for the law of MVD is extensive, the same cannot be said about the literature regarding NOR and the law of MVD in itself. The reference to Planas 3 was maintained because the description of the Law of MVD was originally published by this author in this reference, as shown in Fig. 3 .

## Results

**Fig. 4:**
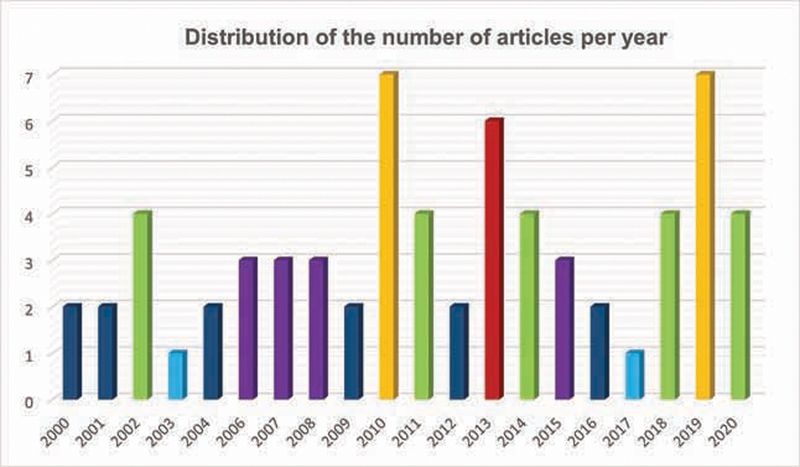
Distribution of articles per year.

Regarding year of publication, there has been relative change over the last two decades, with marked increase in the last decade. This proves that interest in this topic continues to exist, as shown in Fig. 4 .

**Fig. 5:**
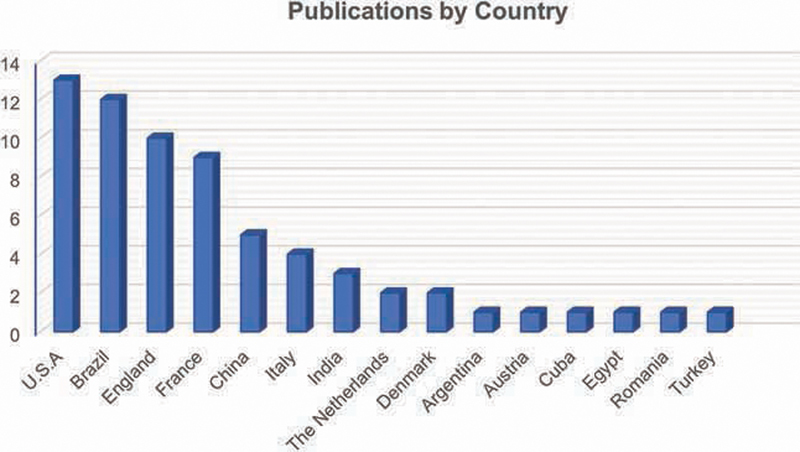
Publications by country.

Fig. 5 shows the high level of scientific interest in the scope in the subject of the present review in some countries.

The articles included in this review were classified according to the impact factors (IFs) of the journals that published them. The IF is a measurement of the journal's relevance, according to international criteria. The level of scientific recommendability of each article was also assessed, following the Oxford Centre for Evidence-based Medicine's classification table ( Table 1 ).

**Table 1:** Recommendation degree (RD) and Impact Factor (IF)

Journal		IF *	RD *
1.	Rev Asoc Argent Ortop Funcional Maxilares 2002; 33(1): 9-25	NA	B
2.	Revista APCD 2019; 73(2):149-154	0.28	C
4.	Rev Orthop Dento Faciale 2001; 35(3):319-336	0.35	B
5.	Case Rep Dent 2013:395784	0.27	C
6.	Rev Orthop Dento Faciale 2002; 36(1):53-73	0.35	B
7.	RFO UPF 24(1):31-37, 29/03/2019	NA	C
8.	Orthodontie Française 2006; 77(1):113-135	0.18	B
10.	Stoma Edu J 2014; 1(2):86-91	1.10	B
11.	Rev Odontol da Univ de São Paulo 2008 Jan-Abr; 20(1):82-86	NA *	B
12.	Journal of Research in Dentistry 2018; 6(6):132-137	NA	B
13.	Eur J Dent 2015; 9(4):573-579	0.59	B
15.	West China Journal of Stomatology 2013; 31(4):331–340	0.11	B
16.	RSBO 2007 (Impr.); 4(2):61-64	NA *	C
17.	Am J Orthod Dentofacial Orthop 2010; 137(4):454.e1-454.e9	1.38	B
18.	Angle Orthod 2014; 84(6):939-945	1.22	B
19.	Head Face Med 2013; 9:42	0.46	B
20.	Am J Orthod Dentofacial Orthop 2004; 126(5):549-554	1.14	B
21.	Compend Contin Educ Dent 2020; 41(4):e1-e6	0,29	B
22.	Int J Orthod Milwaukee 2013; 24(2):21-28	0.06	C
23.	Acta Odontol Scand 2016; 74(2):103-107	1.67	C
24.	Braz Dent J 2010; 21(4):351–355	NA	B
25.	Braz Oral Res 2011; 25(5):446-452	0.71	C
26.	J Int Med Res 2019; 47(5):1908-1915	0.77	B
27.	Chin J Dent Res 2000; 3(1):34-39	0,46	B
28.	Revista APCD 2019; 73(2):102-105	NA	D
29.	Journal of the Lins Dentistry School 2015; 25(1):67-77	0.85	C
30.	Eur J Orthod 2011; 33(6):620-627	0.89	B
31.	Orthod Fr 2006; 77(4):431-437	0.18	B
32.	Am J Orthod Dentofacial Orthop 2008; 134(5):602.e11	1.42	B
33.	J Oral Rehabil 2013; 40(1):69-79	1.93	B
34.	J Prosthodont 2020; 10.1111/jopr.13307	1.23	B
35.	Orthod Fr 2002; 73(2):199-214	0.08	B
36.	Journal of Sichuan University Medical Science Edition 2013; 44(2):231–236	0.17	B
37.	Am J Orthod Dentofacial Orthop 2016; 150(1):140-152	2.20	B
38.	Progress in Orthodontics 2014; 15(1):41	0.44	B
39.	Am J Orthod Dentofacial Orthop 2016; 149(1):46-54	2.20	B
40.	Cranio 2018; 36(3):143-155	1.09	B
41.	Oral Dis 2013; 19(4):406-414	2.37	B
42.	J Esthet Restor Dent 2019; 31(6):620-626	1.78	B
43.	Acta Odontol Scand 2010; 68(6):368-376	1.41	B
44.	Rev Orthop Dento Faciale 2017; 51(3):399-412	0.27	B
45.	Sci Rep 2019; 9(1):15599	3.99	C
46.	Am J Orthod Dentofacial Orthop 2007; 131(4):464-472	1.59	C
47.	Am J Orthod Dentofacial Orthop 2000 Nov; 118(5):541-548	0.79	B
48.	Indian J Dent Res 2011; 22(5):654-658	0.28	C
49.	Annali Di Stomatologia 201; 9(1):53-58	NA	C
50.	Revista CEFAC 2007; 9(3):351-357	0.76	C
51.	World Journal of Orthodontics 2002; 3(3):239-249	NA	C
52.	Brazilian Oral Research 2010; 24(2):204-210	0.90	B
53.	Rev Orthop Dento Faciale 2001; 35(3):339-346	0.35	B
54.	Revista Cubana de Estomatología 2015; 52(2):150-159	0.14	B
55.	Orthod Fr 2006; 77(1):87-99	0. 20	B
56.	Orthod Fr 2010; 81(3):189-207	0.18	B
57.	Arch Oral Biol 2009; 54(2):101-107	1.46	B
58.	Am J Orthod Dentofacial Orthop 2008; 133(6):804-808	1.68	B
59.	Arch Oral Biol 2014; 59(12):1316-1320	1.73	B
60.	Dental Press J Orthod [online] 2020; 25(5):44-50	0.94	C
61.	Indian J Dent Res 2012; 23(6):719-725	0.28	B
62.	J Stomat Occ Med 2009; 2(3):122-130	NA	B
63.	Am J Orthod Dentofacial Orthop 2003; 123(3):329-337	0.83	B
64.	West China Journal of Stomatology 2011; 29(1):48–52	0.11	C
65.	Eur J Orthod 2004; 26(1):65-72	0.97	B
66.	Minerva Stomatologica 2001; 50(7-8):247–263	0.37	C
67.	J Indian Soc Pedod Prev Dent 2010; 28(1):30-33	0.61	B
68.	Progress in Orthodontics 2010; 11(1):53-61	0.19	B

Articles 3, 9 and 14 are not included in the table, as they were not included in the methodology.

* NA: Not Available; IF: Impact Factor; RD: Recommendation Degree.

Out of the 68 abstracts selected, only 65 were used within the methodology. Among the three articles excluded, in two cases this was done to avoid biases, given that they were authored by the first author of the present review, although they were cited only in the introduction and in the discussion sections. The third reference was excluded from the methodology because it was not an indexed article. This was the book by Planas, 3 which was used in this review because it was crucial to the introduction of our review, given that the law upon which our article was based was put forward by that author.

## Discussion

Even though we are fully aware that articles pertaining to clinical cases have little scientific relevance, these were also included in this review, considering that they provide valuable data on the growth and development of various kinds of occlusion and skeletal structures, through analysis of longitudinal data, especially with regard to the functional significance of OP.

These data, in turn, are crucial for understanding the etiology, diagnosis, and treatment of malocclusion. 32 Nonetheless, the theoretical and descriptive elements were cross-referenced with direct results from clinical practice. Among those studies, 64% earned a B-grade recommendation according to the Oxford table, thus demonstrating their relative level of scientific rigor.

In this review, although Brazil is listed in the publications-by-country chart ( Fig. 3 ) as the country with the second largest number of published articles, 60% of the Brazilian publications did not have IF information available, and the remainder presented low IF numbers, below 1.0. On the other hand, the United States was first regarding the quantity of published articles and 100% of its publications had an IF that was freely available, and 70% of them had an IF greater than 1.0.

The country that was third most represented in published articles was the United Kingdom, and 100% of its publications had IF information available and 50% of them had an IF better than 1.0. This shows that English-language publications are indeed at an advantage with regard to having more citations.

On the other hand, in developing countries like Brazil, where universities, research, and scientific journals have been instituted much more recently, these journals tend to have less international visibility and low IFs.

In addition, 90% of all studies that have directly referenced the law of MVD were published in Latin American and French journals. It is unquestionable that these are factors that have restricted wider popularization of the NOR principles. The conclusions of the non-English articles show a high degree of positive correlation and support the scientific basis of the NOR.

The cusp-fossa contact is the typical standard of upper and lower tooth occlusion. At MIP, the inclination of the dental cusps plays a role in distributing occlusal forces in various directions, thus avoiding excessive point pressure. 33 However, several anthropological studies corroborate the notion that, in an attempt to create an ideal occlusion, too much emphasis has been placed on cusp-fossa relationships, with regard to both natural and artificial teeth. Nevertheless, for millions of years, the stomatognathic system has undergone evolutive adaptations in which occlusion suffered strong masticatory stress, thus causing pronounced occlusal and interproximal attrition.

The erosion of dental cusps through attrition erodes occlusal interference as well, thus leading to formation of horizontal occlusal planes and enabling a physiological FO in which the CO coincides perfectly with the MIP. 6 9 On the other hand, a CO-MIP discrepancy may lead to a change in jaw positioning, such that this is therefore a predisposing causal factor for malocclusion. There is even evidence that Angle's classification will change, for many patients, when it is recorded at CO, and this record is a potentially significative diagnostic finding. 34

It is worth pointing out that large CO-MIP discrepancies can also be a contributory factor for the development of TMJ alterations and, in some cases, may lead to dislocation of the articular disc. 14 19 21 35 36

In Ishizaki et al, 17 a study about examining morphological characteristics, occlusal scheme, functional behavior, and deviation from the median line and posteroanterior cephalograms, there is a suggestion that reduction of the height of the dentition on one side leads to lateral jaw adaptation, with contralateral (asymmetrical) dislocation of the condyles. That, in turn, leads to lateral movement of the condyles during functional motion. The posterior OP on the dislocated side was markedly steeper than on the nondislocated side.

An upward inclination of the OP has been associated with jaw deviation in the same direction. An inclination of the OP may cause a vertical discrepancy, which in turn may lead to development of malocclusion. 37 38 39 40

On the side with the larger OP inclination, the occlusal strength and contact area are significantly larger, and TMJD symptoms such as lateralized articular noises (especially in adult patients) are present more often. 39 40 41 42 This is likely to be related to the fact that the jaw is the primary growth center. Consequently, the condylar processes constantly undergo asymmetrical remodeling as a response to the ongoing stimuli of the jaw movements. An asymmetrical jaw function alters the intra-articular mechanical dynamics and leads to persistent activity in one or both condyles.

Patients with jaw asymmetries can therefore show that the morphology and bone density of the condyles on the deviated side differ from what is seen on the nondeviated side. This indicates that the link between asymmetrical jaw function and joint remodeling may lead to TMJ dysfunction. 20 40 43

According to the principles of NOR, occlusion is the result of neuromuscular control over the masticatory system. 44 Neuromuscular activity, in turn, is under the influence of the dental contacts. To achieve better masticatory efficiency, the occlusal plane needs to be modulated throughout one's life, so as to enable free sliding jaw movement, with as many physiological dental contacts as possible. 35

Lila-Krasniqi et al 13 reported that occlusal relations presenting premature contacts caused a shift in jaw closure, and that the condyles could dislocate to reach a maxillomandibular relationship at the MIP and avoid premature contact. Such deviation or dislocation of the condyles may cause a discrepancy between the CO and the MIP and lead to TMJ-affecting occlusal alterations. 43

Therefore, the impact caused by condyle dislocation on the morphology of the condylar processes and in dynamic occlusal function can form a risk factor for the development of TMJ dysfunctions. The conclusion of that study was that there was no statistically significant difference between CR and MIP in the group without TMJD symptoms. The same was not true for the group with symptoms, which presented differences between CR and MIP.

Likewise, Čelar et al 45 in a study using magnetic resonance imaging to produce three-dimensional data for the condylar points at MIP and CR, found concentric condyle positions at MIP, along with considerable variation in condyle position after bi-manual manipulation and use of neuromuscular techniques. The results indicate that differences between these jaw positions due to muscular asymmetries, chewing patterns, and facial asymmetry lead to alterations in the intra-articular spaces, thus confirming the hypothesis that going against law of MVD may lead the TMJ to be functionally and/or morphologically compromised.

In crossbite cases with jaw atrophy, postural jaw deviations and occlusal interference (premature contact) are created. 46 Consequently, CO becomes an uncomfortable position and there is lateralization of the jaw after closure, in an attempt reach a more comfortable position. The effect of such deviation is modification of jaw posture and therefore a significant difference between CR and MIP, which is a crucial characteristic of functional crossbite. 27 47 The cross side becomes the MIP side (the MVD side) and thus preferred chewing side. This leads to asymmetrical growth and alters the normal development of the face. 48 49 50

As in previous reports, we can infer that there will often be modification to condylar positioning due to jaw deviation on the midsagittal plane. 51 In such cases, there are always changes to the activity of the muscles involved in mastication: lips, cheeks, suprahyoid, and infrahyoid. 52 Mastication tends to be unilateral (on the cross side) and there are significant changes to the occlusal plane, thus perpetuating the functional atrophy and malocclusion. 53 54

Dominant unilateral mastication syndrome has been described, consisting of an association of conditions such as asymmetrical muscle activity, deviated jaw, preferential chewing on the deviated side, ascending occlusal plane angled toward the chewing side and TMJ asymmetries. 55 56 Bilateral chewers have markedly better masticatory performance than unilateral chewers. 24 There is strong correlation between the occlusal contact area and the preferred chewing side, which suggests that the MIP is the position where the chewing force is more highly focused. 26 57 Such descriptions are entirely consistent with the law of MVD.

Regarding the stability of functional orthodontic and orthopedic treatments, Rilo et al 58 assessed several occlusal parameters in a group composed of adults with uncorrected posterior crossbite. Their results showed that 64% of the subjects shifted the medial line of the MIP to the crossbite side, and that the lateral orientation angle at the frontal plane was smaller on the crossbite side. Their conclusion was that unilateral posterior crossbite is a malocclusion that, if not corrected in childhood, tends to cause permanent asymmetry.

Likewise, Rovira-Lastra et al 59 observed a significative positive correlation between the preferred chewing side (the side with better masticatory performance) and the asymmetry index. Both conditions interfered directly with the stability of functional orthodontic and orthopedic treatments.

He et al. 27 in a study on changes on occlusion and condyle positioning between CR and MIP positions, reported that nearly all patients presented CR-MIP differences in the three spatial planes and that when the jaw shifted from CR to MIP, the overbite deepened. On the other hand, Abuabara et al 16 and Limme, 8 who followed NOR concepts according to which chewing should be bilaterally alternating and PMFA should be well-balanced, found that developmental atrophies stemming from a deep bite could be resolved through vertical development and normalization of the OP inclination, both of which are necessary conditions for a stable treatment.

Historically, OP has been compared with several other craniofacial reference lines by many authors. In NOR, Camper's plane is considered to be the one that is most suitable for use, which is based on individual fixed cranial structures. OP can present varying inclinations in the sagittal, coronal, or transverse directions, relative to Camper's plane. 60

The MVD side will be located on the side with the larger OP inclination, which is the side with the largest convergence between the OP and Camper's plane. 28 From this, Venugopalan et al 61 used cephalometric studies to analyze the parallelism between Camper and occlusal planes. In their analysis, there was variation in the tragus (the line-orienting point in the Camper plane), at three different heights, named points A, B, and C. In that study, A ran through a higher line toward the midpoint of the tragus, B ran toward the center of that line and C, toward a lower line.

In comparing the relationship between those lines and the OP, however, we can infer that in Angle's class II malocclusions, the posterior occlusal plane (POP) tends to converge with Camper's plane, thus reducing the VD in that (posterior) region. In Angle's class III malocclusions, the same POP tends to diverge to reduce the VD in the anterior region. For Angle's class I, the occlusal plane tends to be parallel to Camper. 62 63 Indeed, it seems as if an inclination of the OP can cause a vertical discrepancy, 43 thus suggesting a possible correlation between the jaw's POP inclination and its position, consistent with causal studies for various malocclusion cases.

Therefore, a broader etiological approach, based on the OP inclination, should be considered in dealing with malocclusion, 32 keeping in mind that an assessment of the PMFA is quite useful in that regard. From the information above, and given that in preferential chewing the working side is always the one with the smallest VD, it can be concluded that in cases of distocclusion, the jaw will take a more posterior position, while in cases of mesiocclusion, it will take up a more anterior position, including during chewing, 64 thus creating a negative feedback cycle that worsens the malocclusion even further. 54

Correction of class II or III malocclusions achieved through use of functional orthodontic or orthopedic devices that aim to correct the OP inclination by seeking parallelism between it and Camper's plane and a bilaterally balanced VD and, as a consequence, bilaterally balanced PMFAs as well, has shown much more stable treatment results. 37 65 66 67 68

Although the reference line that Coro at al 37 used in their report was the Frankfort's plane, they concluded that the POP shows significant correlation with jaw posture. The steeper the POP is, the more retrognathic and hyperdivergent the jaw posture is. The flatter the POP is, the more prognathic and hypodivergent the jaw is. The direction of the lateral jaw deviation is consistent with the POP inclination on that same side, which suggests the possibility of rotational dislocation of the jaw toward the side with the lesser VD.

## Conclusion

Noncompliance with the Law of MVD has been shown to be a predisposing factor for relapse in functional orthodontic and orthopedic treatments, as well as a causal factor in malocclusion and in functional and morphological TMJ dysfunctions.

Based on the articles selected, we can conclude that discrepancy between CO and MIP may lead to a deviation or dislocation of the jaw toward the region, where the maximum of intercusp contacts is established (i.e., the MVD position) and, in most cases, the preferred chewing side. This jaw dislocation will change the OP inclination, which in turn may lead to a change in the maxillo-mandibular relationship and in the position of the condyles within the articular cavity, thus confirming what is stated in the law of MVD.

Observing the PMFA is an effective diagnostic and prognostic method in treatments that aim to normalize the OP, given that the overall balance of the VD can be assessed through using those angles.

This study has shown that the law of MVD is a concept that is both modern and crucial to good clinical practice in all dental specialties dedicated to restoring and/or maintaining the functional balance of the stomatognathic system. Although the majority of the articles used in this study provided a scientific basis for the Law of MVD, there is a need for more specific scientific research to establish the physiological laws that guide and substantiate NOR.
